# Factors affecting reporting of patient safety incidents in the Eastern Cape primary health care

**DOI:** 10.4102/phcfm.v18i1.4993

**Published:** 2026-01-28

**Authors:** Patiswa Tolobisa, Nellie Naranjee, Shamila Moonsamy

**Affiliations:** 1 Department of Nursing, Faculty of Health Sciences, Durban University of Technology, Durban, South Africa

**Keywords:** patient safety, incidents, incident reporting, healthcare standards, positive work environments, primary health care, clinical audits

## Abstract

**Background:**

There is a low and erroneous rate of patient safety incident reporting system in the primary health care institutions in the study. These gaps are identified through clinical audits of patient files, performance reviews and complaints received through the provincial call centre.

**Aim:**

The study aimed to explore and describe factors influencing the reporting of patient safety incidents in primary health care facilities.

**Setting:**

The study was conducted in the Mqanduli District of the Eastern Cape Province. Five healthcare facilities were included, as these were the facilities where the identified problems were evident.

**Methods:**

A qualitative, exploratory, descriptive design was used. Purposive sampling was used to select 10 nurses who were interviewed. Data were analysed by thematic analysis, and measures to ensure trustworthiness, and ethical principles were followed.

**Results:**

The reporting process for patient safety is influenced by a number of factors, such as nurses’ reluctance to report for fear of punishment, a lack of training and education and fear of lawsuits. Nurses need support from management in the form of training and provision of resources, creating a positive work environment and safety culture by not punishing those who make errors and rewarding those who report patient safety incidents.

**Conclusion:**

Nurses receive minimal support from managers, have inadequate knowledge of patient safety incidents (PSI) reporting and guidelines, insufficient resources and high staff workloads, which need to be addressed in order to improve PSI reporting. Nurses require a supportive work environment, with encouragement from colleagues, management and the Department of Health.

**Contribution:**

Recommendations are provided for nursing education, research and practice to enhance nurses’ understanding and proficiency with PSI reporting, thereby ensuring quality of nursing care and patient safety.

## Introduction

Patient safety incidents (PSIs) are a global concern, significantly impacting healthcare delivery and patient outcomes. According to the South African Department of Health, a ‘patient incident’ refers to any unanticipated or unplanned event that could have resulted in or did result in harm to a patient while receiving care, excluding harm because of the patient’s underlying condition or disease progression.^1^ The PSIs can be classified as near misses, no-harm incidents or harmful incidents, necessitating thorough investigation and documentation to identify contributing factors and prevent recurrence.^2^

Reporting PSIs is crucial for improving healthcare safety and preventing future incidents. Effective PSI reporting identifies safety concerns and guides risk-mitigation strategies.^3^ Similarly, Tarkiainen et al.^4^ state that PSI reporting enhances patient care by fostering a culture of learning from incidents and near misses. However, underreporting remains a challenge, with Kihlberg^5^ noting that failure to report increases recurrence, hinders the development of a safety culture and reduces opportunities for systemic improvement. Nurses play a vital role in patient safety, serving as a critical link between patients and healthcare professionals.^6^

The World Health Organization (WHO) has actively promoted PSI reporting to enhance patient safety. In 2005, the WHO’s World Alliance for Patient Safety developed initial standards for adverse event reporting, enabling global knowledge-sharing.^7^ The WHO also introduced a Minimal Information Model for event reporting systems to standardise reporting across different income levels.^1^ Despite these efforts, significant variations exist in reporting rates across nations, with some struggling to implement effective mechanisms.^8^

Barriers to PSI reporting include unfriendly reporting systems, a lack of trust in management, uncertainty regarding consequences and fear of professional repercussions.^9^ Internationally, underreporting is prevalent. In the United States, prescription errors are a leading contributor to patient harm reporting approximately 100 000 suspected medication errors annually.^10^ This contributes to 7000 to 9000 deaths annually.^11^ Similarly, 50% to 96% of medication errors go unreported because of organisational dysfunction, fear of blame and time-consuming reporting processes.^12^

In the United Kingdom, mandatory adverse event reporting was introduced in 2010 alongside a voluntary system, yet reporting rates remain low because of lack of encouragement, repeated reports without action and time constraints.^13^ In Canada, incident reporting systems are underutilised, with physicians often concealing serious events while nurses bear the burden of reporting.^14^ Factors such as a lack of feedback, fear of exposure and limited experience with reporting systems further discourage incident disclosure.

The National Department of Health (NDOH) in South Africa has worked to standardise PSI reporting. The National Guidelines for Patient Safety Incident Reporting and Learning (version 2) were revised to provide clear guidelines on reportable incidents and facilitate the effective use of the national web-based reporting system.^1^ The Ideal Clinic Realisation and Maintenance programme was also introduced to improve healthcare quality and promote PSI reporting compliance.^15^ However, Maphumulo and Bhengu^16^ highlight persistent quality improvement challenges, including avoidable patient deaths because of healthcare system failures.

Patient safety incidents remain prevalent in South Africa, with over 40% of incidents going unreported because of assumptions that non-harmful errors do not require documentation and fears of legal consequences.^17^ In the eThekwini District, KwaZulu-Natal, distrust in PSI reporting, inconsistent PSI definitions, a lack of training and user-unfriendly reporting systems contribute to low reporting rates.^18^ Additional barriers include time-consuming processes, inadequate financial resources and limited system usability.^19^

Despite reinstating clinical governance mechanisms, Gauteng has not seen significant improvements in PSI reporting. Research indicates an increase in adverse events from 4170 in 2019 to 4700 in 2020, with approximately 12 000 patients harmed in public hospitals during the same period.^20^ Similarly, the Western Cape faces challenges such as inconsistent investigations, poor event management and unsupportive leadership.^21^

South Africa’s extensive human immunodeficiency virus (HIV) programme, with over 6.2 million patients receiving antiretroviral therapy necessitates accurate reporting of adverse drug reactions and unexpected serious events to ensure antiretroviral therapy safety.^22^ However, 51.3% of doctors and 54% of nurses fear reporting adverse drug reactions.^23^

In South Africa, the government has introduced various initiatives to improve PSI reporting. In the OR Tambo District, Eastern Cape, a web-based reporting system was established across all primary health care (PHC) facilities to capture and analyse PSIs. However, a persistent issue is the underreporting of medication errors, particularly related to HIV treatment, which are often identified through patient file audits but remain undocumented in official PSI reporting systems. Complaints about missed medications or incorrect prescriptions are typically reported through the provincial quality assurance call centre rather than designated PSI reporting channels. South Africa has adopted the WHO’s Minimal Information Model, mandating healthcare providers to report any unexpected incidents that jeopardise patient safety.^1^

The King Sabata Dalindyebo (KSD) sub-district in the Eastern Cape of South Africa presents a case in point. Despite implementing a web-based PSI reporting system in 2018, reporting rates in KSD’s PHC facilities remain low. Medication errors, particularly in managing HIV-positive patients, are frequently identified during patient file audits but not captured in the official reporting system. This highlights challenges in reporting processes and significant knowledge gaps within the healthcare workforce.

This study aimed to explore the factors influencing PSI reporting within the KSD sub-district, focusing on nurses’ perspectives. The specific objectives were to: (1) explore factors affecting PSI reporting in PHC facilities in KSD sub-district and (2) provide recommendations to enhance PSI reporting practices.

By identifying these factors, the study sought to inform strategies for improving the accuracy and completeness of patient safety data, thereby enhancing patient outcomes and contributing to the creation of a safer healthcare environment. Understanding the factors that hinder effective reporting is crucial for developing targeted interventions that can improve the culture of safety in PHC facilities and ensure the proper management of patient care incidents. Through this exploration, the study aspired to offer insights that can support the broader goal of improving healthcare quality in South Africa and beyond.

## Research methods and design

### Study design

A qualitative, exploratory and descriptive design was employed to explore factors influencing the reporting of PSIs in PHC facilities from the perspectives of nurses. This allowed the researchers to obtain a detailed understanding of the issue and gather information based on first-hand experiences, which is rich, authentic and original.

### Setting

Mqanduli is a cluster within the KSD sub-district in the province of the Eastern Cape. There is one district hospital, one community health centre and eight PHC clinics. A total number of 63 nurses work in PHC clinics in the cluster under study. The study was conducted in five out of eight healthcare facilities within the designated region. These specific sites were selected because they were where the problem under investigation had been initially identified by the researcher. The purposive sampling approach ensured that the study focused on contexts where the issue was known to exist, allowing for more meaningful and relevant data collection. However, this method may introduce selection bias, as the findings reflect the experiences and practices of facilities already affected by the problem and may not be generalisable to all healthcare settings. The results should therefore be interpreted within the context of these selected facilities.

### Population and sampling

The target population consisted of 48 nurses employed full-time in PHC facilities within the selected cluster. A purposive sampling technique was used to select 20 participants. Five nurses participated in the pilot study, and 15 were scheduled to participate in the actual study. However, data saturation was reached by the 10th participant. The final sample of 10 participants included four operational managers, five registered nurses and one enrolled nursing assistant (ENA). The inclusion criteria required nurses to be permanently employed by the Department of Health for at least 3 years, be 18 years or older and be willing to participate. The exclusion criteria included nurses employed on a temporary or contractual basis, those with less than 3 years of experience and those unwilling to participate or unavailable during data collection.

### Data collection

An interview guide containing open-ended questions and follow-up probes was used to obtain detailed information from the participants. A pilot study was conducted with five nurses to test the interview guide and the data collection process. The data collection process was conducted through semi-structured interviews, guided by two central research questions: what factors influence PSI reporting in PHC facilities in KSD sub-district and what recommendations can be made to improve PSI reporting? In-depth, face-to-face interviews with consenting nurses were conducted by the primary researcher. Each interview lasted approximately 20 min – 30 min and were supplemented with field notes to enrich the data.^24^ To ensure confidentiality and minimise disruptions, a consulting room within each PHC clinic was used for the interviews. A ‘No Entry Please’ sign was placed outside the door to prevent interruptions. The interviews were conducted in English as all nurses preferred to be interviewed in English. Probing questions were utilised, as needed, to gain deeper insights from participants. With the permission of the participants, interviews were audio-recorded to ensure accuracy, while field notes were taken to document non-verbal cues and the researcher’s reflections. Data saturation was achieved by the 10th participant, as no new information emerged beyond this point. This marked the conclusion of the interview process, ensuring a comprehensive exploration of the research objectives.

### Data analysis

Data were analysed using Braun and Clarke’s^25^ thematic analysis approach. The researchers first familiarised themselves with the data by listening to recordings, reading transcripts multiple times and identifying patterns. Initial codes were generated based on commonalities, which were then grouped into potential themes and sub-themes. A thematic map was created, and themes were refined, reviewed and renamed where necessary.

The final analysis for the research questions resulted in three themes, which were validated through consensus meetings with an independent coder. Audio recordings were transcribed verbatim, and multiple listening sessions ensured accuracy. An inductive coding approach was used, and themes were linked to identity work tactics found in existing literature.

### Reflexivity

The researchers maintained a reflexive approach throughout the study to acknowledge and minimise potential biases. Personal experiences, assumptions and preconceptions were continuously examined to ensure they did not influence data interpretation. A reflexive journal was kept to document thoughts and reflections during data collection and analysis. Peer discussions and collaboration with an independent coder further enhanced the credibility and trustworthiness of the findings.

### Trustworthiness

The study adhered to Lincoln and Guba’s four trustworthiness criteria: credibility, transferability, dependability and confirmability.^26^

Credibility was ensured through prolonged engagement, where the researcher spent sufficient time with participants to build trust and gain deeper insights. Member checking was conducted, allowing participants to verify the accuracy of their transcriptions. Triangulation strengthened the study by incorporating multiple data sources, including interviews, field notes and audio recordings. In addition, supervisor validation was performed, with the research supervisor cross-checking the transcriptions against raw data to ensure accuracy. Transferability was established by providing detailed descriptions of the research setting, participant selection and data collection methods, enabling readers to determine the applicability of the findings to similar contexts. Data collection continued until theoretical saturation was reached, ensuring a comprehensive understanding of PSI reporting behaviours. Dependability was maintained by offering a thorough description of the research process, allowing future researchers to replicate the study. An audit trail was kept, documenting key research decisions and independent coder meetings were held to ensure consistency and reliability in the data analysis process. Confirmability was achieved by ensuring that findings were based on participants’ experiences rather than researcher bias. Reflexive practices were employed, and participants were contacted after transcription to verify the accuracy of their responses, further strengthening the objectivity of the study.

### Ethical considerations

Ethical clearance to conduct this study was obtained from the Eastern Cape Department of Health (EC-202310_008) and the Durban University of Technology Research Ethics Committee (No. 116/23). The study adhered to key ethical principles to ensure the protection of participants’ rights and well-being. All processes in the study adhered to the 1964 Helsinki Declaration, ethical standards of the institution and research committees. Authorisation letters from the district and sub-district managers permitted the study to proceed. Participation was entirely voluntary, with no coercion or consequences for refusal. Participants were fully informed about the study’s purpose, risks, benefits and their right to withdraw at any time. Written informed consent was obtained, and participants had the opportunity to ask questions before agreeing to take part. The researcher ensured that participants were not harmed or exploited. Confidentiality was maintained, and all interview transcriptions and recordings were securely stored. Efforts were made to minimise harm and maximise benefits, and participants did not exhibit distress during data collection. Participants’ rights to self-determination and full disclosure were upheld. They had the freedom to decline participation, refuse to answer questions or withdraw at any time. No undue inducements were offered, and confidentiality was strictly maintained, with clinic and participant names kept anonymous. Participants’ identities remained protected, with names and institutional details encrypted. Code numbers were assigned, and identifying information was stored separately from collected data. The researcher upheld ethical obligations to maintain confidentiality and anonymity, ensuring no third-party access without explicit consent. Fair and equal treatment was ensured for all participants, regardless of age, experience or nationality. Inclusion and exclusion criteria were applied consistently, and participants were not pressured to disclose information they were uncomfortable sharing. Their identities remained confidential, with coded identifiers used to protect privacy.

## Results

Participants’ sociodemographic data regarding characteristics of the study participants were collected before each interview session. The findings of these data were quantified to facilitate interpretation and better understanding. From a gender point of view, participants comprised eight females and two males. All participants were above 30 years of age. Participants comprised four operational managers, five professional nurses and one ENA. All the participants had 5 years or more working experience in the PHC facilities. Two participants have a degree in Nursing, one participant has a certificate in nursing, three had a postgraduate Diploma in Nursing and four had a Diploma in Nursing (see Table 1).

**TABLE 1:** Participants’ demographics.

Site	Participant code	Gender	Category	Age (in years)	Work experience	Qualification
A	A1	Female	Operational Manager	Above 50	More than 10 years	Degree in Nursing
A	A2	Female	Professional nurse	41–50	More than 10 years	Post Graduate Diploma in Nursing
B	B3	Male	Operational Manager	Above 50	More than 10 years	Degree In Nursing
B	B4	Female	Enrolled Nursing Assistant (ENA)	41–50	More than 10 years	Certificate
C	C5	Female	Operational Manager	41–50	More than 10 years	Post Graduate Diploma in Nursing
C	C6	Male	Professional nurse	31–40	5–10 years	Diploma in Nursing
D	D7	Female	Operational Manager	41–50	More than 10 years	Post Graduate Diploma in Nursing
D	D8	Female	Professional nurse	41–50	5–10 years	Diploma in Nursing
E	E9	Female	Professional nurse	41–50	5–10 years	Diploma in Nursing
E	E10	Female	Professional nurse	41–50	More than 10 years	Diploma in Nursing

The study’s results reveal several factors contributing to PSIs in healthcare facilities. The findings are categorised into three key themes: healthcare worker contributory factors, patient contributory factors and clinic setting contributory factors. Each theme is supported by direct participant quotes. To ensure confidentiality, each participant was assigned a unique code, which is presented alongside their gender (Male or Female), nursing category (Professional Nurse: P; Operational Manager: OM; Enrolled Nursing Assistant: ENA) and work experience (WE) in years.

### Theme 1: Healthcare worker contributory factors

The study identified several healthcare worker-related factors contributing to the failure to report PSIs. These factors include non-adherence to clinical guidelines, errors in prescription verification, inadequate communication among staff members and negligence in prioritising patient care. Participants provided various accounts highlighting these issues, demonstrating the impact on patient safety.

Participants observed that staff did not adhere to triage guidelines because of long queues, which led to delays in patient care:

‘According to the guideline, we are supposed to prioritise the patient according to their conditions. We didn’t prioritise because of the long queue outside.’ (C6, Male, PN, 3–5 years WE)

Errors in documentation were another significant issue. Incomplete patient records, including missing signatures, were identified as contributing to the lack of accountability in PSI reporting:

‘… [*A*]nd there was no signature and also that a gap that was identified because you can’t just write on the card without a signature.’ (D7, Female, OM, 7–10 years WE)

Furthermore, improper patient file retrieval led to the administration of discontinued treatments, compromising patient safety:

‘At the registration office, the first file was retrieved and the patient was given the treatment that was stopped.’ (D8, Female, PN, 3–5 years WE)

Delayed diagnosis because of failure to detect symptoms in a timely manner was also reported, illustrating the severity of these errors:

‘Child was missed to be seen that she has measles until she had severe symptoms after 2 days.’ (B3, Male, OM, 7–10 years WE)

Ineffective communication among staff members was a recurrent theme, with poor prescription verification leading to medication errors:

‘… [*T*]he communication was poor and another thing the staff didn’t check the prescription before so it was the staff error.’ (C5, Female, OM, 7–10 years WE)

Additionally, concerns were raised regarding healthcare professionals operating beyond their scope of practice, as defined by the South African Nursing Council (SANC). Instances were reported where junior nurses and ENAs performed tasks they were not authorised to undertake:

‘The complaint … the junior nurse wrote on the patient card and then in my understanding she did not know that she was not supposed to write on patient card, only a professional nurse is allowed to write there.’ (D7, Female, OM, 7–10 years WE)

‘It is done by ENAs who are not informed; they have no insight of what to do immediately on the cases of maternity cases.’ (C6, Male, PN, 3–5 years WE)

The absence of senior personnel further exacerbated the situation, leaving junior staff members unsupported and vulnerable to making critical errors:

‘The clinician was a community service nurse who was working alone in the absence of senior personnel.’ (B3, Male, OM, 7–10 years WE)

Participants identified a lack of knowledge about what constitutes a PSI and which incidents should be reported:

‘We are not clear about PSI to be reported and categories of severity; if the PSI is not life-threatening at times we ignore and do not report it. Fear to be blamed is also a barrier and distrust of reporting system.’ (E10, Female, PN, more than 10 years WE)

Fear of punishment rather than receiving support during the PSI reporting process was another critical factor discouraging incident documentation:

‘You cannot be sure if you are getting support or you getting punishment because during the PSI management when you are doing the report it always feels like you are being blamed more than being supported.’ (C5, Female, OM, 7–10 years WE)

In addition, there was a lack of awareness regarding the availability of reporting systems, such as computer-based tools for capturing near misses:

‘I was not aware that there is a computer system that captures near misses.’ (B4, Male, PN, 6–10 years WE)

A lack of exposure to PSIs and insufficient training on reporting procedures led to uncertainty about the correct reporting processes:

‘The staff member was not exposed to the incident before so we didn’t know normal frame to report.’ (C6, Male, PN, 3–5 years WE)

‘Some of the PSIs we don’t know really which one to report and which one not to report.’ (A1, Female, OM, 7–10 years WE)

Workplace pressure was highlighted as a major barrier to PSI reporting. Because of staffing shortages and high patient volumes, healthcare workers often struggled to recall proper reporting protocols:

‘The pressure that we are working under, we were short-staffed, and also we didn’t have the recent in-services to remember what steps to take.’ (A2, Female, PN, 7–10 years WE)

‘… [*W*]orking under a lot of pressure and the reporting period is sometimes short.’ (C5, Female, OM, 7–10 years WE)

Excessive patient loads further contributed to stress and hindered thorough incident reporting:

‘We see more than normal, for example, if we supposed to see 30 per nurse, we end up seeing 40 so we manage them having a pressure that there are lot of people outside.’ (C6, Male, PN, 3–5 years WE)

Healthcare worker-related factors significantly contribute to the failure to report PSIs. These factors include errors in clinical practice, poor communication, a lack of adherence to guidelines, inadequate documentation and scope-of-practice violations. Furthermore, knowledge gaps, fear of blame, distrust in the reporting system and high work pressure further impede PSI reporting. Addressing these challenges through targeted interventions, such as training, awareness programmes and systemic improvements, is essential to fostering a culture of patient safety and improving incident reporting practices.

### Theme 2: Patients’ contributory factors

Patient-related factors played a significant role in the occurrence of PSIs. Participants highlighted that many patients lacked health literacy, which affected their ability to recognise early symptoms and seek timely medical attention. As a result, some patients delayed seeking care, often presenting at healthcare facilities at advanced stages of illness. This delay hindered early diagnosis and timely intervention, potentially leading to worsened health outcomes.

One participant emphasised the impact of this delay, stating:

‘They don’t have the knowledge on when to come to the clinic, what to do when they feel or experience any illnesses. Some of the patients come to the facility at a later stage, some of them are coming for antenatal clinic in the later stage of their pregnancies, so we are unable to pick those illnesses at the early stage.’ (C6, Male, PN, 3–5 years WE)

Another significant concern was the continuation of discontinued treatments because of miscommunication or failure to review prescriptions. Some patients unknowingly continued using medications that had been stopped, increasing the risk of adverse effects.

A participant highlighted this issue, recalling an incident where a patient unknowingly continued an outdated treatment:

‘The patient went home with the treatment that was stopped a long time ago.’ (D8, Female, PN, 3–5 years WE)

Cultural and traditional beliefs also influenced patient health-seeking behaviours. Some patients delayed seeking medical care because they first consulted traditional healers, which often resulted in worsened health conditions by the time they arrived at healthcare facilities.

One participant voiced concern over this trend, stating:

‘Some patients come late to the clinics; they consult traditional healers first.’ (E9, Female, PN, 3–5 years WE)

Another participant elaborated on the complexities of cultural influences:

‘Patients sometimes believe that their condition is caused by ancestral spirits or curses, so they try traditional remedies before coming to the clinic. By the time they arrive, their condition has worsened.’ (B4, Male, PN, 6–10 years WE)

### Theme 3: Clinic setting contributory factors

The healthcare environment was identified as a critical factor affecting patient safety. Participants expressed concerns about the lack of essential resources, including medical supplies and medications, which hindered effective patient management.

A participant explained:

‘… [*A*]nd also the drugs that are always not available, so those are also the most causes of patient safety incidents.’ (A1, Female, OM, 7–10 years WE)

Reporting challenges were also identified. The participants shared that it becomes difficult for them to report the PSIs when there are barriers in the work environment that hinder them from doing so. They indicated that data, Internet and phones are examples of these barriers:

‘… [*P*]oor network connectivity, clinic doesn’t have phones and Wi-Fi.’ (E9, Female, PN, 3–5 years WE)

‘The major challenge is the network, the password.’ (A1, Female, OM, 7–10 years WE)

Infrastructure deficiencies, such as inadequate consultation rooms and small rooms for child health, created safety hazards.

One participant described unsafe conditions:

‘The child health room is very small to accommodate a firm and flat surface to put the scale, like a table or a counter. Scale was put on top of a couch which is not level, making the scale lose balance.’ (E10, Female, PN, 7–10 years WE)

‘The contributory factors were one, I think the infrastructure is the problem because we are not doing these vitals on the same consulting room where the midwife will be able to see and act immediately …’ (E10, Female, PN, more than 10 years WE)

Transportation issues also played a role:

‘The clinic is about 85-kilometre gravel road away from town.’ (E9, Female, PN, 3–5 years WE)

Participants found that environmental hazards posed risks to patient safety:

‘There is a long grass with weeds; the patient saw a snake getting inside her bag.’ (B4, Female, ENA, 7–10 years WE)

Another participant recounted:

‘A snake that falls on top of the patient.’ (A2, Female, PN, 7–10 years WE)

Overcrowding also posed a problem:

‘The clinic is overcrowded; there is no space for filing.’ (D8, Female, PN, 3–5 years WE)

Participants overwhelmingly highlighted the shortage of healthcare personnel as a major barrier to ensuring patient safety. Understaffing led to increased workloads, stress and the potential for errors.

One participant observed:

‘The shortage of staff … because when you are short staffed you are also in a risk of mismanaging the patients.’ (C6, Male, PN, 3–5 years WE)

Another participant added:

‘We don’t have staff because we are short staffed, we are overwhelmed. They ended up not reporting.’ (A2, Female, PN, 7–10 years WE)

The absence of senior personnel also affected care delivery:

‘… [*S*]hortage of staff because the clinician was a community service nurse who was working alone in the absence of senior personnel.’ (B3, Male, OM, 7–10 years WE)

Participants mentioned third parties such as trade unions in the reporting of PSI. Trade unions seem to influence the reporting system of PSI in the health facilities. This influence causes a negative influence as it encourages healthcare workers not to perform as required. According to the participants, when certain staff members are asked to write a statement so that management can have a complete account and act, they report the incident to the unions because they fear they may be fired. The unions then tell the staff members not to write anything, so they fail to report the incident because no information is available.

Participants added:

‘There is a tendency of reporting to the unions by health care workers. Unionist will say that they must not talk to us as managers and we ended up leaving it and not investigating the incident and not capture it.’ (D8, Female, PN, 3–5 years WE)

‘They wanted to consult their unions because they said whenever they were told by the unions that whenever they have to write a statement the union must be involved.’ (D7, Female, OM, 7–10 years WE)

‘Nurses refuse to write comments because their trade unions warn them against doing so for fear of losing their jobs.’ (C5, Female, OM, 7–10 years WE)

Workplace pressure was highlighted as a major barrier to PSI reporting. Because of staffing shortages and high patient volumes, healthcare workers often struggled to recall proper reporting protocols:

‘The pressure that we are working under, we were short-staffed, and also we didn’t have the recent in-services to remember what steps to take.’ (A2, Female, PN, 7–10 years WE)

‘… [*W*]orking under a lot of pressure and the reporting period is sometimes short.’ (C5, Female, OM, 7–10 years WE)

Excessive patient loads further contributed to stress and hindered thorough incident reporting:

‘We see more than normal for example if we supposed to see 30 per nurse we end up seeing 40 so we manage them having a pressure that there are lot of people outside.’ (C6, Male, PN, 3–5 years WE)

## Discussion

The study identified multiple healthcare worker-related factors contributing to the failure to report PSIs. These include non-adherence to clinical guidelines, prescription verification errors, inadequate communication among staff and negligence in prioritising patient care. Failure to follow triage guidelines because of long queues led to delays in patient care, compromising patient safety. Documentation errors, such as incomplete records and missing signatures, created accountability gaps in PSI reporting. In addition, the retrieval of incorrect patient files resulted in the administration of discontinued treatments, jeopardising patient well-being. Delayed diagnosis, including a missed measles case, further highlighted the impact of reporting failures.

Ineffective communication among healthcare workers was a recurring issue, with poor prescription verification leading to medication errors. Junior nurses and ENAs were sometimes found performing tasks beyond their scope of practice, violating SANC regulations. The absence of senior personnel exacerbated these challenges, leaving inexperienced staff unsupported and prone to errors. A significant concern raised by participants was the lack of clarity on what qualifies as a PSI and which incidents require reporting. Many healthcare workers were unsure about the severity categories, with some choosing to ignore non-life-threatening incidents. Fear of blame and punitive measures further discouraged reporting, making PSI documentation seem more punitive than supportive.

Moreover, some healthcare workers were unaware of electronic reporting systems, such as computer-based tools for capturing near misses. Limited exposure to PSIs and insufficient training on reporting protocols contributed to uncertainty. High workplace pressure, staff shortages and excessive patient loads hindered proper reporting procedures. Because of overwhelming patient volumes, nurses struggled to allocate time for documentation, increasing the likelihood of unreported PSIs.

Participants acknowledged that while some actively reported PSIs, others refrained because of various barriers. Workplace challenges included high service delivery pressure, resource shortages, insufficient infrastructure and limited technological support, such as poor Internet access. Agoro et al. highlight that unreliable or expensive internet access impedes optimal electronic pharmacovigilance reporting in Kenya,^27^ which is similar to findings by Gqaleni and Mkhize advocating for revised implementation strategies and in-service training to improve PSI reporting adherence.^28^ This study recommends targeted education and training programmes to enhance healthcare workers’ proficiency in PSI reporting.

Another significant challenge was the influence of third parties, such as trade unions, on PSI reporting. Some participants perceived unions as discouraging adherence to required reporting protocols. O’Brady and Doellgast support this, noting that unions can restrict managerial discretion and obstruct the introduction of new technology, affecting productivity.^29^ Heavy workloads further contributed to increased errors. Salama et al. observe that excessive workloads lead to stress, causing a loss of focus and raising the risk of errors.^30^ Banda, Simbota and Mula emphasise that inadequate nurse-patient ratios compromise patient safety, as nurses struggle to manage multiple cases simultaneously.^31^

A culture of negativity and blame within healthcare facilities also discouraged PSI reporting, leading nurses to conceal errors from supervisors and colleagues. Management’s control over contract renewals and staff oversight significantly influences employee behaviour.^32^ To address this, healthcare institutions should foster a supportive environment through effective communication, just culture principles and constructive feedback mechanisms. Debriefings and learning-focused discussions can empower healthcare professionals to disclose PSIs without fear of retribution.

In addition, participants highlighted a lack of feedback after incident reporting and uncertainty regarding what should be reported as barriers to effective PSI documentation. Establishing a positive work environment prioritising patient safety is essential. Healthcare institutions should ensure adequate staffing, implement improved recruitment processes and maintain appropriate nurse-patient ratios. Furthermore, healthcare workers should have access to sufficient resources and tools to facilitate PSI reporting.

Beyond organisational factors, patient-related issues also influenced PSI reporting. Some nurses hesitated to report PSIs caused by patient behaviours, such as delays in seeking care or adherence to traditional beliefs, perceiving them as unavoidable. Fear of blame or backlash deterred nurses from reporting incidents related to patient actions, as they worried about scrutiny from management. Heavy workloads and time constraints further discouraged reporting, as managing patients with advanced illnesses left little time for additional documentation.

The normalisation of late patient presentations led nurses to view them as routine rather than reportable events. Additionally, a lack of clear guidelines on reporting patient-driven incidents created uncertainty. To bridge this gap, healthcare facilities must provide explicit guidelines on reporting PSIs related to patient behaviours, ensuring nurses understand the importance of comprehensive documentation. Establishing a non-punitive reporting culture where nurses feel safe to disclose incidents is crucial. Enhancing patient health literacy through education initiatives can also help reduce preventable PSIs caused by delays in seeking care or mismanagement of treatment.

Addressing these barriers will encourage comprehensive PSI reporting, leading to better patient safety interventions and improved healthcare outcomes. Establishing a culture of accountability and continuous improvement in PSI reporting will protect patients while supporting nurses in delivering safer, more effective care.

### Strengths and limitations

This study offers a nuanced and in-depth exploration of the barriers to PSI reporting in public healthcare settings. A major strength lies in the use of qualitative methods, which allowed for the collection of rich, context-specific data that may not have been effectively captured through quantitative approaches. Through semi-structured interviews with healthcare workers, the study uncovered complex and multifaceted challenges, such as staff perceptions, fear of blame, communication breakdowns, inadequate supervision, union involvement and the influence of institutional culture.

The study employed structured thematic analysis, which enhanced the rigour and credibility of the findings by ensuring that emergent themes were clearly identified and systematically interpreted. Furthermore, the research took a holistic view of PSI reporting, examining internal facility dynamics and broader systemic influences. By shifting the focus away from individual fault and towards systems-level issues, the study provides actionable insights for improving healthcare policy, patient safety systems and clinical practice. Recommendations such as strengthening infrastructure, improving access to technology, promoting a just culture and investing in targeted education and training emerged directly from participant experiences.

Data saturation was achieved after conducting ten interviews suggesting that the sample size was adequate for the exploratory aims of the study. Nevertheless, the possibility exists that additional nuances could have been uncovered with a broader participant pool. The reliance on self-reported data presents a limitation, as participants may have underreported or overreported their experiences because of fear of reprisal or social desirability bias.

The study was conducted in a specific geographical location within a resource-constrained public health system, which may influence the nature of the reported barriers. Therefore, while the findings are not intended to be generalised in a statistical sense, they may be transferable to other similar contexts in the province, across South Africa, or in comparable international settings. Thick descriptions of the healthcare environment, participants’ roles and institutional structures are provided to support the reader in assessing the potential transferability of these findings.

Resource constraints also limited the inclusion of a wider range of institutions, and the focus was primarily on healthcare workers’ perspectives. Despite these limitations, the study contributes significantly to understanding the systemic challenges of PSI reporting in the public sector and provides a foundation for further research and policy development.

### Recommendations

#### Education and training

Gqaleni and Mkhize suggest revising the implementation strategy in conjunction with routine in-service training for healthcare professionals to successfully promote and encourage adherence to PSI reporting standards.^28^ Therefore, in order to enhance healthcare workers’ understanding and proficiency with PSI reporting, this study recommends education and training. Healthcare professionals ought to participate part in these programmes.

#### Improve communication

Develop effective communication channels between staff members, management and patients so as to maintain positive support, facilitation and better coordination. This should be taken as a number one priority in order to improve PSI reporting.

#### Establish positive patient safety culture

Create a just and faultless culture that empowers healthcare professionals to disclose PSIs without hesitation, enabling them to grow from their mistakes. In order to guarantee that learning and practice improvement occur, supervisors ought to conduct debriefings and provide feedback.

#### Improve work environment

Establish positive redress in the physical work environment and actively encourage patient safety as a top priority within healthcare organisations. A positive work environment can bring success when sufficient staff is allocated. Ensuring the right nurse-patient ratio and streamlining the hiring process will allow for adequate time for PSI reporting. It is important to have a supportive and encouraging work environment for nurses that recognises their efforts and encourages them, so they feel comfortable reporting PSIs. Staff members should have access to sufficient resources and the tools they need to report PSIs.

#### Recommendations for future research

Following the results of this study, further research could investigate factors influencing PSI reporting by nurses on a larger scale across the nation. Furthermore, PHC providers in other districts, private hospitals, districts and tertiary hospitals should be included in future studies. Future studies should focus on the needs of the patient in order to get input from those who use the healthcare system.

## Conclusion

The underreporting of PSIs in PHC settings is influenced by a range of interrelated factors. Key barriers include knowledge gaps among healthcare workers, high workloads, inadequate infrastructure, poor technological resources and trade union involvement. Fear of blame, a lack of feedback and the absence of clear reporting guidelines further discourage incident reporting. Patient-related factors, such as delays in seeking care and cultural beliefs, also contribute to the challenge.

Addressing these issues requires a multifaceted approach: education, improved infrastructure, clear reporting protocols and the establishment of a supportive, non-punitive reporting culture. Targeted training programmes, better staffing levels and enhanced technological resources are essential in overcoming these barriers. Creating an environment that promotes transparency, accountability and continuous improvement will ensure healthcare workers are empowered to report PSIs, ultimately improving patient safety and healthcare outcomes.
